# Neurodegenerative disease-associated inclusion bodies are cleared by selective autophagy in budding yeast

**DOI:** 10.1080/27694127.2023.2236407

**Published:** 2023-08-07

**Authors:** Austin Folger, Chuan Chen, Marie-Helene Kabbaj, Karina Frey, Yanchang Wang

**Affiliations:** a Department of Biomedical Sciences, College of Medicine, Florida State University, Tallahassee, FL, USA; b College of Biological Sciences, Hebei University, Baoding, Hebei, China; c Department of Biological Sciences, Florida State University (undergraduate student), Tallahassee, USA

**Keywords:** Autophagy, IBophagy, mutant huntingtin, aβ42, inclusion body

## Abstract

Protein misfolding, aggregation, and accumulation cause neurodegenerative disorders. One such disorder, Huntington’s disease, is caused by an increased number of glutamine-encoding trinucleotide repeats CAG in the first exon of the huntingtin (*HTT*) gene. Mutant proteins of Htt exon 1 with polyglutamine expansion are prone to aggregation and form pathological inclusion bodies in neurons. Extensive studies have shown that misfolded proteins are cleared by the ubiquitin-proteasome system or autophagy to alleviate their cytotoxicity. Misfolded proteins can form small soluble aggregates or large insoluble inclusion bodies. Previous works have elucidated the role of autophagy in the clearance of misfolded protein aggregates, but autophagic clearance of inclusion bodies remains poorly characterised. Here we use mutant Htt exon 1 with 103 polyglutamine (Htt103QP) as a model substrate to study the autophagic clearance of inclusion bodies in budding yeast. We found that the core autophagy-related proteins were required for Htt103QP inclusion body autophagy. Moreover, our evidence indicates that the autophagy of Htt103QP inclusion bodies is selective. Interestingly, Cue5/Tollip, a known autophagy receptor for aggrephagy, is dispensable for this inclusion body autophagy. From the known selective autophagy receptors in budding yeast, we identified three that are essential for inclusion body autophagy. Amyloid beta peptide (Aβ42) is a major component of amyloid plaques found in Alzheimer’s disease brains. Interestingly, a similar selective autophagy pathway contributes to the clearance of Aβ42 inclusion bodies in budding yeast. Therefore, our results reveal a novel autophagic pathway specific for inclusion bodies associated with neurodegenerative diseases, which we have termed IBophagy.

## Introduction

Proper protein folding is essential for the functions of all proteins. Although protein folding is tightly regulated, protein misfolding can occur under normal physiological conditions and is exacerbated by genetic mutations, environmental stress, and oxidative stress. Misfolded proteins are prone to aggregation, resulting in the formation of small soluble aggregates and large insoluble inclusion bodies (IBs) [1]. Misfolded proteins confer cytotoxicity by disrupting multiple cellular processes or by depleting free ubiquitin [2-4]. Accumulation of misfolded proteins is linked to more than 40 different neurodegenerative disorders, including Alzheimer’s and Huntington’s diseases [5]. To combat the cytotoxicity of misfolded proteins, cells have developed quality control systems to correctly refold or dispose of misfolded proteins. The ubiquitin-proteasome system and autophagy play a major role in the clearance of misfolded proteins [6-8]. However, the autophagic clearance of misfolded protein aggregates and IBs is not fully understood.

Huntington’s disease is a neurodegenerative disorder characterised by progressive motor and cognitive deficits caused by an expansion of CAG (glutamine-encoding) repeats within the exon 1 of the *HTT* gene. Individuals who have more than 39 CAG repeats will develop symptoms of Huntington’s disease, and the age of disease onset is inversely correlated with the number of CAG repeats [9]. It is well established that the accumulation of N-terminal fragments of Htt with polyQ repeats gives rise to cytotoxicity [10]. Aberrant splicing contributes to the generation of pathogenic Htt exon 1, which comprises 17 N-terminal amino acids followed by the polyQ tract and a proline-rich region [11]. Results using a mouse model indicate that expression of Htt exon 1 with polyQ expansion (hereafter mHtt) is sufficient to cause the development of Huntington’s disease [12]. Thus, this mHtt has been widely used to investigate its cytotoxicity and clearance.

A recent study analysed the structural and material properties of mHtt assemblies *in vivo* and *in vitro*. The polyQ and proline-rich region of mHtt protein drive reversible liquid-like assemblies. The liquid-like assemblies can convert into solid- or gel-like assemblies that contain fibrillar structures both *in vitro* and in yeast/mammalian cells [13]. Yeast and mammalian cells expressing mHtt form IBs, and high resolution cryo-electron tomography shows that the core structure of mHtt IBs consists of amyloid fibrils [14,15]. In addition to the mHtt fibrils, these IBs also include different cellular proteins and membranous organelles. The surface of mHtt IBs is enriched in endoplasmic reticulum (ER) membrane, mitochondria, endomembranes, and vesicles [16]. Therefore, expression of pathogenic mHtt first leads to the formation of small and soluble aggregates (oligomers) [3,17]. These aggregates further facilitate generation of mHtt amyloid fibrils and the subsequent formation of IBs, which are large and insoluble [14,16]. Although soluble mHtt aggregates can disrupt multiple cellular pathways by interacting with various proteins [3], mHtt IBs are also cytotoxic since the fibril protrusions from the IBs can reorganise the ER network and reduce membrane dynamics [14]. Therefore, the clearance of both misfolded protein aggregates and IBs is important for cells to combat the cytotoxicity of pathogenic mHtt proteins.

Macroautophagy (hereafter referred to as autophagy) is a highly conserved cellular process that degrades and recycles cellular components and proteins [18]. Autophagy can be selective or non-selective. Starvation-induced autophagy is non-selective since it randomly engulfs cytosolic components into autophagosomes, which subsequently fuse with the vacuole/lysosome for degradation [19]. In contrast, selective autophagy utilises selective autophagy receptors (SARs) to engulf specific cargos, which are then degraded after autophagosome-vacuole/lysosome fusion [20]. Autophagy plays an important role in the clearance of misfolded proteins, and deletion of core autophagy-related genes leads to accumulation of polyubiquitinated inclusions/aggregates in mouse neurons [21,22]. Moreover, SARs, such as p62, OPTN, and ALFY localise at mHtt aggregates and are likely involved in their autophagic clearance [23-25]. In addition, a conserved SAR Cue5/Tollip promotes the autophagy of misfolded protein aggregates, including mHtt, in both yeast and human cells [26]. Thus, an important open question is whether misfolded protein aggregates and IBs share the same autophagy pathway for their clearance.

Amyloid β (Aβ) peptides are generated by proteolytic processing of a transmembrane amyloid precursor protein, and Aβ42 is a major component of extracellular amyloid plaques in the brains of patients with Alzheimer’s disease [27]. Expression of Aβ42-GFP from a galactose-inducible promoter in yeast cells results in the formation of intracellular inclusions [28,29]. Thus, budding yeast has been used as a model to study the aggregation and toxicity of Aβ42 [30,31]. Lewy bodies containing α-synuclein are a neuropathological hallmark of Parkinson’s disease. Expression of α-synuclein in budding yeast also leads to formation of inclusions [32,33]. However, the role of autophagy in the clearance of IBs formed by Aβ42 and α-synuclein remains poorly understood.

Our previous works show that IBs formed by Htt exon 1 with 103 polyQ (Htt103QP) can be cleared by autophagy as evidenced by the defective vacuolar delivery of Htt103QP-GFP in *atg8∆* mutants [34,35], but the role of other autophagy-related genes in the autophagic clearance of Htt103QP IBs remains unknown. In this study, we constructed a series of mutants lacking various autophagy-related genes and examined Htt103QP IB autophagy. First, we found that the core autophagy-related genes are required for the autophagy of Htt103QP IBs. Second, our results indicate that selective autophagy is responsible for the clearance of Htt103QP IBs. Lastly, we identified three SARs responsible for Htt103QP IB clearance by autophagy: Atg36 (pexophagy), Atg39 (nucleophagy), and Atg40 (ER-phagy), supporting the idea that mHtt IBs hijack these SARs for their autophagy. Interestingly, IBs formed by Aβ42, but not α-synuclein, are also cleared by the similar autophagy pathway. Therefore, our results identified an autophagy pathway specific for IBs formed by some neurodegenerative disease-associated proteins in budding yeast, and we refer to this pathway as IBophagy.

## Results

### The core autophagy machinery is required for autophagic clearance of mHtt IBs

Autophagy has been implicated in the clearance of mHtt aggregates in both yeast and mammalian cells [26,36,37]. Although our previous works showed autophagic clearance of mHtt IBs [34,35], the autophagy pathway for mHtt IBs remains obscure. We first quantitatively analysed the process of autophagic clearance of Htt103QP IBs in wild-type (WT) and *atg8Δ* mutants since Atg8 is essential for different types of autophagy [38]. For mHtt IB autophagy analysis, we utilised a previously constructed integrating plasmid *P_GAL_-FLAG-Htt103QP-GFP* [34], which contains the first exon of Htt with a 103-polyQ expansion and a proline-rich domain (hereafter Htt103QP). In this construct, Htt103QP is tagged with FLAG at the N-terminus and green fluorescent protein (GFP) at the C-terminus and is under the control of a galactose-inducible promoter (GAL). *P_GAL_-FLAG-Htt103QP-GFP* was integrated into the yeast genome. These yeast cells also harbour mApple-tagged Vph1 (Vph1-mApple) to mark the vacuole and *pep4Δ* to stabilise Htt103QP inside the vacuole, as Pep4 is the key vacuolar protease [39,40].

**Figure 1.**
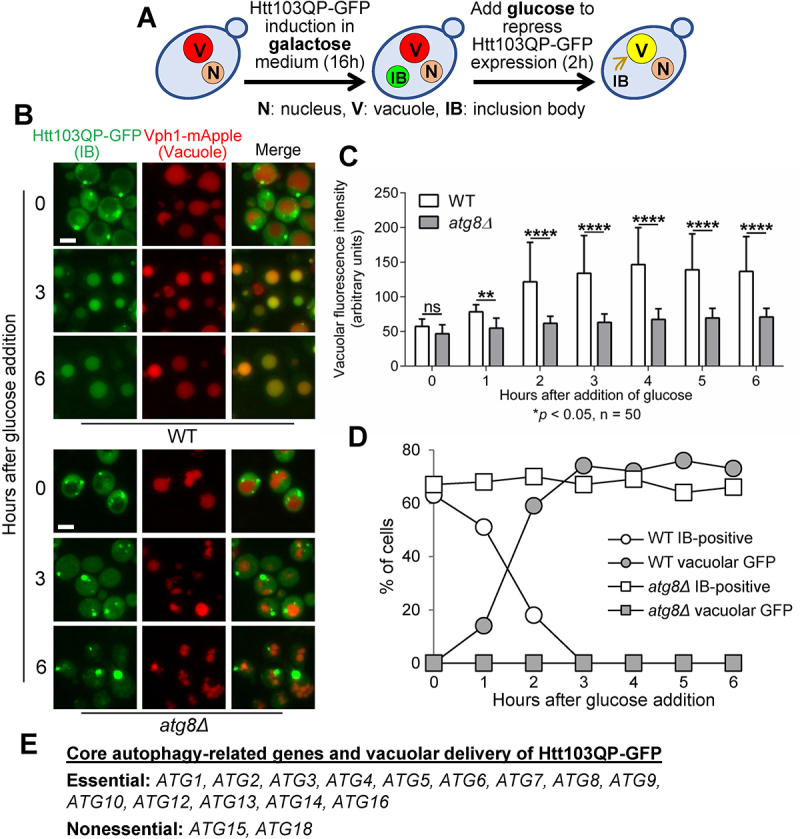
**Htt103QP IBophagy requires the core autophagy machinery. a)** A diagram showing the protocol for IBophagy induction. Yeast strains with *P_GAL_FLAG-Htt103QP-GFP* were grown in 30°C galactose medium for 16 hours to induce Htt103QP IB formation. We then added glucose and hydroxyurea to repress Htt103QP expression and arrest the cell cycle, respectively. Vacuolar localisation of Htt103QP-GFP indicates IBophagy. **b)** Htt103QP IBophagy in WT and *atg8Δ* cells. After IBophagy induction, the localisation of Htt103QP-GFP was examined over time. Vph1-mApple marks the vacuole. Here, we show the images of Htt103QP-GFP and Vph1-mApple before (0) and after IBophagy induction for 3 and 6 hours. Scale bar = 5 μm. **c)** The IBophagy in WT and *atg8Δ* mutant cells was quantified by measuring fluorescence intensity of Htt103QP-GFP inside the vacuole in 50 cells at each time point. Statistical significance was determined by *p < 0.05, using Sidak’s two-way ANOVA. **d)** IBophagy quantification in WT and *atg8Δ* mutants was done by counting the percentage of cells with either an IB or vacuolar GFP signal (n > 100). **e)** The requirement of core autophagy-related genes for Htt103QP IBophagy.

We induced Htt103QP IB formation by growing cells in galactose media at 30°C. After overnight growth (16 hours), most cells showed a single large GFP dot, indicating Htt103QP IB formation. Then, glucose was added to suppress Htt103QP expression, which induces autophagy. At the same time 200 mM hydroxyurea was added to arrest cells in S-phase and eliminate the potential effect of cell division on autophagy (Figure 1a). After glucose addition for 2 hours, a large portion of WT cells showed GFP signal inside the vacuole, a likely result of autophagic trafficking of Htt103QP-GFP into the vacuole. However, no obvious vacuolar GFP signal was observed in *atg8Δ* mutant cells even after glucose addition for 6 hours (Figure 1b). We further measured the GFP fluorescence intensity inside the vacuole after glucose addition. It was clear that WT cells, but not *atg8Δ* mutants, exhibited a sharp increase of GFP signal after glucose addition for 2 hours, and the difference between WT and *atg8Δ* cells was significant (Figure 1c). We also counted the number of cells with IBs and/or vacuolar GFP. After glucose addition, the number of WT cells with IB(s) decreased over time, but the number of WT cells with vacuolar GFP increased. In clear contrast, these numbers remained stagnant for *atg8Δ* mutant cells (Figure 1d), indicating that the vacuolar delivery of Htt103QP-GFP depends on the autophagy pathway.

To examine if the vacuolar delivery of Htt103QP-GFP is dependent on IB formation, we compared vacuolar GFP intensity in yeast cells expressing either Htt25QP-GFP or Htt103QP-GFP after IBophagy induction. As reported previously, yeast cells expressing Htt25QP-GFP fail to form IBs [41]. The increase of vacuolar GFP intensity after IBophagy induction was much less in cells expressing Htt25QP-GFP compared to that in cells expressing Htt103QP-GFP (Figure S1). The failure of vacuolar delivery of Htt25QP-GFP indicates that mHtt IBs, but not free mHtt proteins, are delivered into the vacuole. We previously showed proteasomal degradation of Htt103QP [2], thus the failure of vacuolar delivery of Htt103QP in *atg8Δ* cells might be a result of impaired proteasomal degradation. To test this possibility, we examined Htt103QP stability using the glucose shut-off assay. The expression of FLAG-Htt103QP-GFP was induced in galactose medium for 1 hour, then glucose was added to shut off its expression. *atg8Δ* cells exhibited similar Htt103QP degradation kinetics as WT cells, while deletion of the E3 ligase *SAN1* significantly stabilised Htt103QP as previously described (Figure S2) [2]. This result indicates that the defective vacuole delivery of Htt103QP in *atg8Δ* cells is not due to impaired Htt103QP proteasomal degradation. Therefore, the defective delivery of Htt103QP IBs into the vacuole as well as the persistent appearance of Htt103QP IBs in *atg8Δ* cells after glucose addition suggests autophagic clearance of Htt103QP IBs.

In budding yeast, AAA ATPase Hsp104 functions as a disaggregase to dissolve protein aggregates and amyloids in cooperation with other chaperones [42]. We previously showed that some chaperone proteins facilitate autophagy of Htt103QP IBs, and observed colocalization of Hsp104 with Htt103QP IBs [35]. To test if Hsp104 contributes to the clearance/autophagy of Htt103QP IBs, we examined Htt103QP-GFP signal in *hsp104∆* strains using our IBophagy protocol. IBophagy was clearly observed in *hsp104∆* cells, although the kinetics is slightly slower than that in WT cells (Figure S3). Therefore, like other chaperone proteins, Hsp104 facilitates IBophagy but it is not essential for this process.

Among the autophagy-related (*ATG*) genes in budding yeast, 16 of them constitute the core autophagy machinery, which is essential for autophagosome formation. These core *ATG* genes are shared by both non-selective and selective autophagy [19]. In addition to *atg8Δ*, we constructed yeast strains expressing Htt103QP-GFP but lacking other core autophagy related proteins (Atg1, Atg2, Atg3, Atg4, Atg5, Atg6, Atg7, Atg9, Atg10, Atg12, Atg13, Atg14, Atg15, Atg16, and Atg18), then Htt103QP IBophagy was examined in these mutants as described above. Strikingly, all these mutants exhibited defective vacuolar localisation of Htt103QP-GFP after glucose addition, except for *atg15Δ* and *atg18Δ* (Figure 1e and S4A, B, and C). Atg18 and Atg21 are homologous proteins that bind phosphoinositides for autophagy-related pathways [43]. Thus, the presence of Atg21 might bypass the requirement of Atg18 for IBophagy. Atg15 is a lipase responsible for dissolution of autophagosome membrane inside the vacuole. In yeast cells lacking Atg15, defective autophagy is likely attributed to the failed degradation of components delivered into the vacuole [44,45].

We found that GFP intensity increased in the vacuole in *atg15Δ* cells over time after IB autophagy induction, but at a significantly delayed rate compared to WT cells. Additionally, more *atg15Δ* cells contained an IB compared to WT cells after autophagy induction, indicating impaired delivery of Htt103QP IBs into the vacuole in *atg15Δ* cells (Figure S4A, B, C). This result contrasts with the efficient autophagic delivery of cargos into the vacuole in *atg15Δ* cells seen in some studies [46-48]. However, other studies showed defective autophagosome delivery to the vacuole in *atg15Δ* mutant cells [49,50], although it is unclear how Atg15 is involved in this vacuolar delivery. We also assessed the sensitivity of autophagy-deficient yeast mutants to Htt103QP expression. It appeared that *atg8∆* cells tolerated Htt103QP overexpression on galactose plates similar to WT cells, but *atg11∆* cells showed slightly slower growth on galactose plates (Figure S4D). Together, our results support the conclusion that all the core autophagy genes except *ATG15* and *ATG18* are essential for Htt103QP IBophagy.

### Selective autophagy is responsible for Htt103QP IB clearance

The autophagy pathway can be selective or non-selective. In contrast to non-selective autophagy that randomly delivers cytoplasmic components into the lysosome/vacuole, selective autophagy utilises selective autophagy receptors (SARs) for the autophagic degradation of specific cellular components [20]. For selective autophagy, scaffold protein Atg11 serves as the adaptor between SARs and the core autophagy-related protein Atg8 [51-55]. In addition, Atg11 recruits core autophagy subcomplex Atg1/Atg13 to initiate autophagy as well as Atg9 vesicles to elongate autophagosome membrane [56,57]. In non-selective autophagy, Atg17 is counterpart of Atg11 for autophagy initiation and membrane elongation. Atg17 exists as part of the Atg17-Atg29-Atg31 complex, which is essential for starvation-induced non-selective autophagy [58].

**Figure 2.**
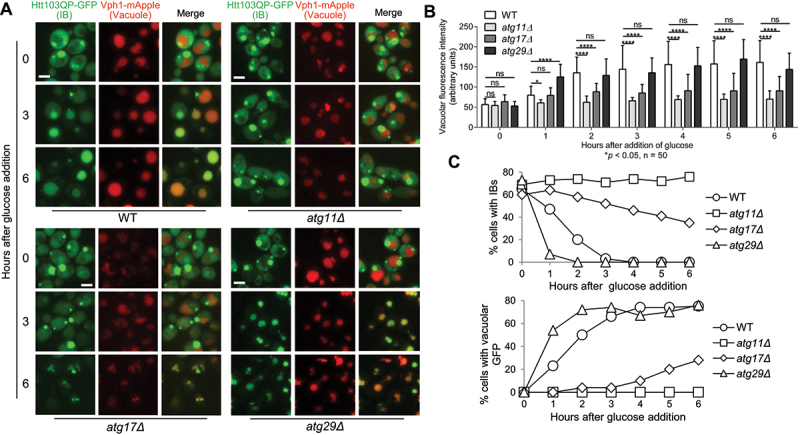
**Htt103QP IBophagy requires the selective autophagy machinery. a)** Htt103QP IBophagy in WT and mutants lacking genes specific for selective or non-selective autophagy. The protocol used was the same as described in Figure 1 for the examination of IBophagy. Here, we show images of Htt103QP-GFP and the vacuole (Vph1-mApple) before and after IBophagy induction. Scale bar = 5 μm. **b)** IBophagy was quantified by measuring GFP fluorescence intensity inside the vacuole in 50 cells at each time point. Statistical significance was determined by *p < 0.05, using Tukey’s two-way ANOVA. **c)** IBophagy was also quantified by counting the percentage of cells with either an IB or diffuse vacuolar GFP signal (n > 100).

To determine whether Htt103QP IBophagy is selective, we analysed the IBophagy process in *atg11Δ, atg17Δ, atg29*Δ, and *atg31Δ* mutants using the protocol described above. We found that this IBophagy was defective in *atg11Δ* mutant cells as evidenced by the absence of vacuolar Htt103QP-GFP signal and the persistent presence of cytoplasmic IBs. In clear contrast, Htt103QP IBophagy was normal in *atg29Δ* and *atg31Δ* as evidenced by the appearance of Htt103QP-GFP inside the vacuole and the disappearance of Htt103QP IBs, but partially defective in *atg17Δ* cells (Figure 2a,b,c and S5). The normal IBophagy in *atg29Δ* and *atg31Δ* cells indicates that Atg29 and Atg31 are dispensable for this process. The partial IBophagy defect in *atg17Δ* cells is consistent with the role of Atg17 in several types of selective autophagy, including piecemeal autophagy of the nucleus, mitophagy, and pexophagy [59-61]. The role of Atg17 in selective autophagy could be attributed to its function in Atg9 trafficking [62,63], but *atg17Δ* cells retain weakened capacity for selective autophagy [60]. Therefore, these results support the idea that Htt103QP IB clearance utilises the selective autophagy pathway.

### The selective autophagy receptor (SAR) for aggrephagy, Cue5, is not required for Htt103QP IBophagy

**Figure 3.**
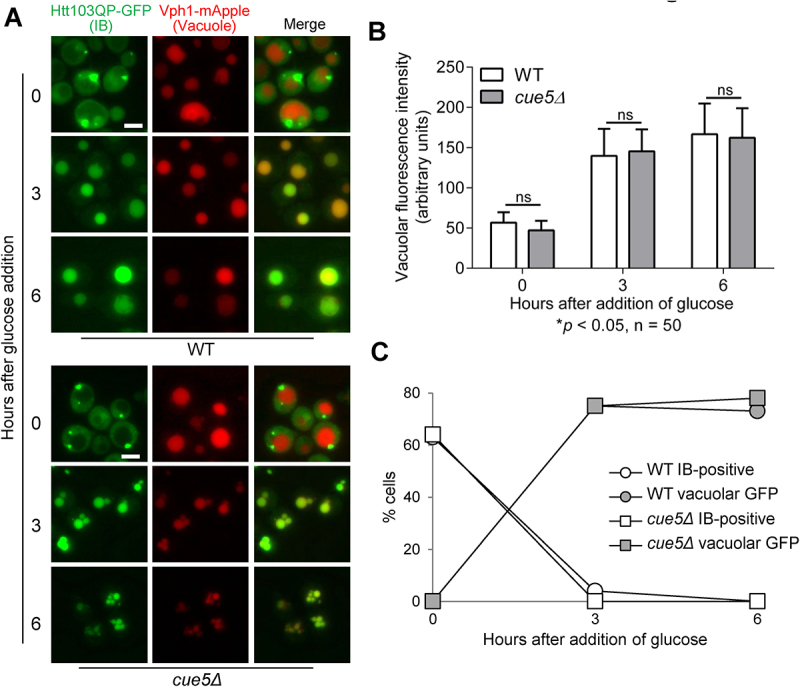
**Yeast aggrephagy SAR Cue5 is not required for Htt103QP IBophagy. a)** IBophagy proceeds normally in *cue5Δ* cells. The IBophagy protocol was the same as described. Here, we show the localisation of Htt103QP-GFP and the vacuole (Vph1-mApple) before and after IBophagy induction. Scale bar = 5 μm. **b)** IBophagy was quantified by measuring GFP fluorescence intensity in 50 cells inside the vacuole over time. Statistical significance was determined by *p < 0.05, using Sidak’s two-way ANOVA. **c)** IBophagy was also quantified by counting the percentage of cells that contained either an IB or vacuolar GFP signal (n > 100).

In budding yeast, SAR Cue5 (Tollip in mammals) is required for the autophagic clearance of misfolded proteins, including mHtt. Cue5 binds to Atg8 and recognises ubiquitinated misfolded protein aggregates using its CUE (coupling of ubiquitin conjugation to ER degradation) domain, which allows engulfment of misfolded protein aggregates by the phagophore [26]. Thus, Cue5 is believed to be the SAR for aggrephagy. We asked whether Cue5 was also involved in Htt103QP IBophagy. Utilising the IBophagy assay described above, we detected efficient delivery of Htt103QP-GFP into the vacuole as well as the disappearance of Htt103QP IBs in *cue5Δ* mutant cells, indicating that Cue5 is not required for Htt103QP IBophagy (Figure 3a,b,c). The different requirement of Cue5 in the autophagic clearance of mHtt proteins is likely due to different autophagy protocols used. Lu et al. utilised a *P_CUP1_Htt-96Q* construct to induce Htt-96Q expression from *CUP1* promotor in yeast cells by adding CuSO_4_, but it was unclear if these yeast cells formed Htt-96Q IBs when the cells were collected for the autophagy assay [26]. In our IBophagy assay, we induced IB formation by growing cells with *P_GAL_Htt103QP* overnight in galactose media before glucose was added to induce IBophagy. The dispensable role of aggrephagy SAR Cue5 in Htt103QP IBophagy indicates that IBophagy is distinct from aggrephagy. Notably, Cue5 is the only known yeast SAR that does not bind to Atg11 during autophagy initiation [20], but we demonstrated the essential role of Atg11 in Htt103QP IBophagy (Figure 2), which further supports the notion that IBophagy is different from aggrephagy.

### Selective autophagy of Htt103QP IBs depends on certain SARs

**Figure 4.**
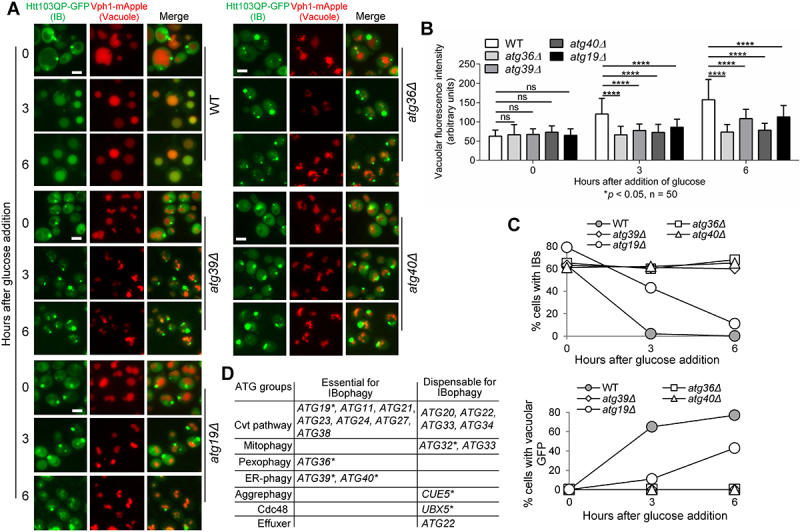
**Htt103QP IBophagy shares SARs with other types of selective autophagy. a)** Defective Htt103QP IBophagy was detected in SAR mutants *atg36Δ, atg39Δ, atg40Δ*, and *atg19Δ*. The IBophagy protocol described above was used to examine IBophagy in WT and SAR mutants. The localisation of Htt103QP-GFP and Vph1-mApple (vacuole) in WT, *atg36Δ, atg39Δ, atg40Δ*, and *atg19Δ* cells before and after IBophagy induction is shown. Scale bar = 5 μm. **b)** Quantification of IBophagy in WT and SAR mutants was done by measuring GFP fluorescence intensity inside the vacuole in 50 cells at each time point. Statistical significance was determined by *p < 0.05, using Tukey’s two-way ANOVA. **c)** The percentage of cells that contained either an IB or vacuolar GFP signal in WT and SAR mutants over time. **d)** The requirement of non-core autophagy-related genes for IBophagy. * marks different types of SARs.

Because IBophagy is selective, SARs should be required for this process. We used our IBophagy protocol to screen the known SAR mutants and found that Atg36, Atg39, and Atg40 were required for Htt103QP IBophagy. In the mutant cells lacking these SARs, Htt103QP IBs persisted in the cytosol and their vacuolar delivery was blocked after IBophagy induction (Figure 4a,b,c). Atg36 is the SAR for pexophagy [52]; Atg40 is for ER-phagy [54,64]; while Atg39 is required for both ER-phagy and nucleophagy [54]. Because ER membrane and endomembrane are abundant in the periphery of mHtt IBs [14,16], one explanation is that mHtt IBs hijack these SARs through IB-associated membrane fragments, which facilitates IBophagy.

Atg32 is the mitophagy receptor [65], and the surface of mHtt IBs is also enriched in mitochondria [16], but we found that Atg32 was dispensable for Htt103QP IBophagy (Figure S6). Recently, Ubx5 has been shown to be the SAR for excess or damaged Cdc48 complexes [66]. Moreover, the Cdc48 segregase is required to alleviate the cytotoxicity of mHtt aggregates by promoting their segregation and the subsequent proteasomal degradation [2]. However, we observed normal Htt103QP IBophagy in *ubx5Δ* mutant cells (Figure S7), indicating that Ubx5 is dispensable for IBophagy.

The cytoplasm-to-vacuole targeting (Cvt) pathway is a selective autophagy that specifically transports hydrolases to the vacuole in yeast cells, and Atg19 is the cargo receptor for this pathway [55]. Using the IBophagy protocol described above, we observed a partial IBophagy defect in *atg19Δ* mutant cells. This was evidenced by the compromised increase of Htt103QP-GFP signal inside the vacuole as well as delayed disappearance of IBs in *atg19Δ* cells compared to WT cells (Figure 4a,b,c). In addition to SAR Atg19, a group of genes is required for the Cvt pathway but not for starvation-induced bulk autophagy. We further examined Htt103QP IBophagy in these mutant cells. Our results showed that Atg21, Atg23, Atg24, Atg27, and Atg38, but not Atg20, Atg22, Atg33, and Atg34, were essential for the vacuolar delivery of Htt103QP IBs (Figure 4d, S8). Therefore, the core autophagy-related genes and some nonessential autophagy-related genes are required for Htt103QP IBophagy, revealing a new pathway specific for the autophagic clearance of IBs.

### The colocalization of IBophagy SARs with Htt103QP IBs

**Figure 5.**
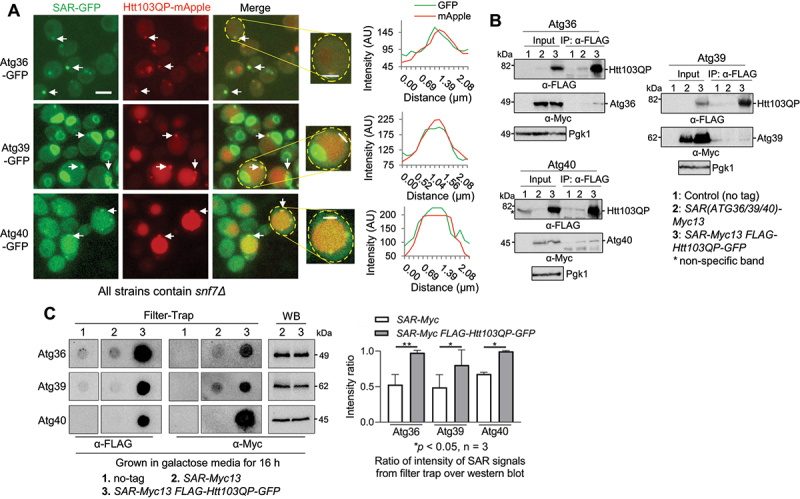
**The SARs essential for IBophagy colocalize with Htt103QP IBs. a)** The colocalization of SARs Atg36, Atg39, and Atg40 with Htt103QP IBs. The SARs were tagged with GFP and their colocalization with Htt103QP-mApple IBs was examined after growth at 30°C in galactose media for 16 hours. The strains used in this experiment contain *snf7Δ*, which prevents autophagosome-vacuole fusion. Here, we show the images for SAR colocalization with Htt103QP IBs (arrows). To analyse the colocalization, a straight white line was drawn through the Htt103QP-mApple. Then SAR-GFP signals and the fluorescence intensity (AU) along the line was plotted using ImageJ (right). Scale bar = 5 μm. **b)** The interaction of SARs with Htt103QP IBs as shown by Co-IP. SARs were tagged with 13Myc in strains with and without *P_GAL_FLAG-Htt103QP-GFP*, and all strains were grown to log phase in raffinose media before galactose addition for two hours to induce Htt103QP expression. SAR enrichment was found in the IP fractions from cells with FLAG-Htt103QP induction after pulldown with anti-FLAG beads, indicating SAR interaction with Htt103QP aggregates. **c)** The association of SARs with Htt103QP IBs using filter-trap assay. SARs were tagged with 13Myc in strains with and without *P_GAL_FLAG-Htt103QP-GFP*, and all strains were grown in galactose media for 16 hours to induce Htt103QP IB formation. Cell lysates were prepared and filtered through a nitrocellulose membrane using a filter-trap apparatus. Anti-FLAG and anti-Myc antibodies were used to detect the Htt103QP and SARs trapped in the membrane. Quantification of the blot was done by measuring signal intensity in each dot. The ratio of trapped SARs over the total SARs was analysed after three repeats. Statistical analysis was determined by *p < 0.05, using Tukey’s two-way ANOVA.

If an SAR is essential for IBophagy, we expect its colocalization with IBs. Therefore, we constructed strains expressing Htt103QP-mApple as well as GFP-tagged Atg36, Atg39, or Atg40. In addition, these strains contain *snf7Δ* to prevent autophagosome-vacuole fusion [67,68], which allows us to visualise SAR localisation in the absence of autophagosome clearance. After induction of Htt103QP-mApple expression by growing the cells in galactose medium for 16 hours at 30°C, we found that majority of Htt103QP IBs showed colocalization with these three SARs (Figure 5a), although the relative intensity of these three SARs varied. The plot of intensity along a line crossing Htt103QP IBs further validates the colocalization of these SARs with IBs (Figure 5a, right) Additionally, all three SARs colocalize with IBs at approximately the same level. This observation supports the notion that Atg36, Atg39, and Atg40 SARs are present in Htt103QP IBs, which is consistent with their role in IBophagy.

We further used co-immunoprecipitation (IP) approach to examine the association of these SARs with Htt103QP. Because Htt103QP IBs are pelleted after centrifugation, we were unable to analyse SAR-IB association using co-IP method. To overcome this problem, we first grew yeast cells with *SAR(ATG36/39/40)-13Myc* or *SAR-13Myc P_GAL_FLAG-Htt103QP-GFP* in raffinose medium and then added galactose to induce FLAG-Htt103QP-GFP expression for 2 hours when no IB formation was detected. Cells were harvested to prepare cell lysates and we examined Htt103QP-SAR interaction using a co-IP method with anti-FLAG antibody beads. Both Atg36 and Atg39 were detected in the IPed fractions from cells expressing FLAG-Htt103QP SAR-13myc, but not from samples expressing only SAR-13Myc (Figure 5b). In contrast, Atg40 was detected in the IPed fractions from cells with or without FLAG-Htt103QP expression, but a clear enrichment was observed in the sample expressing FLAG-Htt103QP (Figure 5b). These results suggest that Atg36/39/40 interact with Htt103QP, but further experiments are needed to clarify if Htt103QP monomers or aggregates interact with these SARs.

We further used a filter-trap assay to analyse the association of SARs with Htt103QP IBs, because this assay detects large, SDS-insoluble aggregates by filtration through a nitrocellulose membrane [69]. *SAR(ATG36/39/40)-Myc13* and *SAR-Myc13 P_GAL_FLAG-Htt103QP-GFP* cells were grown in galactose medium for 16 hours to induce Htt103QP IB formation, and the cell lysates were prepared and subjected to the filter-trap assay. The membranes were then probed with anti-Myc and anti-FLAG antibodies. After normalisation with SAR expression using regular Western blotting with anti-Myc antibodies, we found the significant enrichment of Atg36/39/40 in the filter-trap assay spots of cell lysates with Htt103QP expression (Figure 5c). Together, these results suggest the association of Atg36/39/40 SARs with Htt103QP aggregates/IBs, further supporting the role of these SARs in Htt103QP IBophagy.

### The role of SAR cofactors in Htt103QP IBophagy

**Figure 6.**
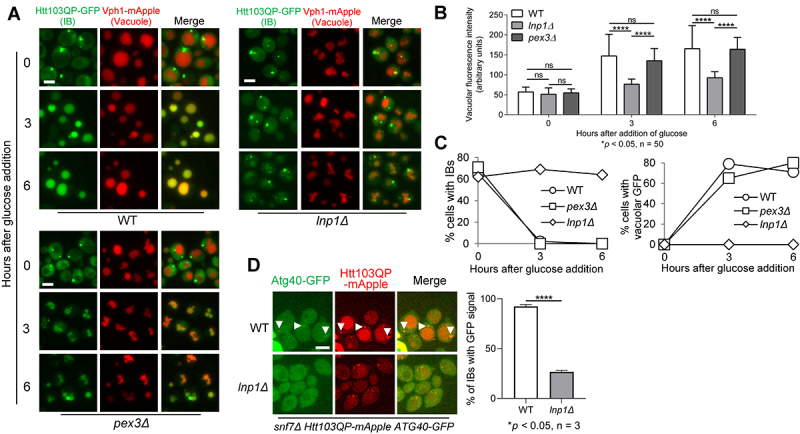
**SAR cofactors and Htt103QP IBophagy. a)** Htt103QP IBophagy in the absence of Atg40 cofactor (*lnp1Δ*) and Atg36 cofactor (*pex3Δ*). The same IBophagy protocol was used for this experiment. Here, we show the localisation of Htt103QP and the vacuole (Vph1-mApple) before and after IBophagy induction. Scale bar = 5 μm. **b)** IBophagy was quantified by measuring GFP fluorescence intensity inside the vacuole in 50 cells at each time point. Statistical significance was determined by *p < 0.05, using Tukey’s two-way ANOVA. **c)** Htt103QP IBophagy was also quantified by counting the percentage of cells that contained either an IB or diffuse vacuolar GFP signal (n > 100). **d)** The colocalization of Atg40 with Htt103QP IBs in the absence of Atg40 cofactor Lnp1. The localisation of Htt103QP-mApple and Atg40-GFP in WT and *lnp1∆* cells (30°C) was examined as described above. The representative images and the percentage of IBs with Atg40 colocalization are shown. Statistical significance was determined by *p < 0.05, using an unpaired t-test.

Some SARs have cofactors to facilitate their subcellular localisation and function. The Atg36 cofactor Pex3 is a peroxisome membrane protein that recruits Atg36 to the peroxisome after its formation, which facilitates pexophagy [52,70]. Lnp1 is an ER membrane protein, which stabilises ER network rearrangements and recruits Atg40 to the autophagy machinery for ER-phagy [71]. To test whether these cofactors are also required for Htt103QP IBophagy, we subjected *lnp1Δ* and *pex3Δ* mutant cells to our IBophagy assay. We found abolished Htt103QP IB delivery to the vacuole in *lnp1Δ* cells, but *pex3Δ* cells showed efficient delivery (Figure 6a). Quantitative analysis exhibited increased vacuolar GFP intensity and decreased IBs in *pex3Δ* cells after IBophagy induction, but not in *lnp1Δ* cells (Figure 6b,c), indicating that Lnp1, but not Pex3, is required for Htt103QP IBophagy. We further examined Atg40-IB colocalization in *lnp1Δ* cells, and we observed abolished colocalization, which is consistent with the essential role of Lnp1 in IBophagy (Figure 6d). Therefore, our results have identified three SARs as well as a cofactor Lnp1 required for IBophagy.

### Inclusion bodies formed by Alzheimer’s disease-associated protein Aβ42 are also cleared by selective autophagy

**Figure 7.**
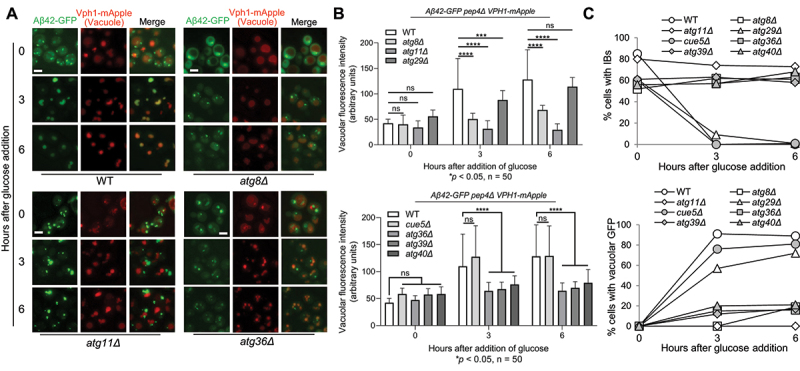
**IBophagy in yeast cells expressing Aβ42. a)** Aβ42 IBophagy in WT and representative autophagy mutants. The protocol used was the same as described in Figure 1 for the examination of Htt103QP IBophagy. Here, we show images of Aβ42-GFP and the vacuole (Vph1-mApple) before and after IBophagy induction. Scale bar = 5 μm. **b)** IBophagy was also quantified by measuring GFP fluorescence intensity inside the vacuole in 50 cells at each time point. Statistical significance was determined by *p < 0.05, using Tukey’s two-way ANOVA. **c)** IBophagy was quantified by counting the percentage of cells with either an IB or diffuse vacuolar GFP signal (n > 100).

In addition to mutated Htt proteins, other neurodegenerative disease-associated proteins, such as Aβ42 and α-synuclein, also form IBs in yeast cells [28,30,32,72]. We first confirmed IB formation in yeast cells expressing these proteins tagged with GFP from a galactose-inducible promoter. Then we analysed their IBophagy after glucose addition as described for Htt103QP IBophagy. Interestingly, vacuole GFP enrichment was detected in yeast cells expressing Aβ42-GFP, but not α-synuclein-GFP (Figure 7a and S9). The vacuolar delivery of Aβ42-GFP was blocked in the absence of the core autophagy gene *ATG8*, indicating that this delivery depends on autophagy. Deletion of selective autophagy gene *ATG11*, but not the starvation specific autophagy-related gene *ATG29*, also blocked vacuolar delivery of Aβ42-GFP, indicating that Aβ42 IBophagy is selective (Figure 7). We further analysed the requirement of SARs for Aβ42 IBophagy. Similar to Htt103QP IBophagy, the absence of Atg36/39/40, but not the aggrephagy receptor Cue5, blocked Aβ42 IBophagy (Figure 7), further indicating that IBophagy is different from aggrephagy. We noticed a slight accumulation of Aβ42-GFP signal in cells lacking Atg8, Atg36, Atg39, and Atg40 after glucose addition (Figure 7c), which might be attributed to an autophagy-independent mechanism. Together, these results support the conclusion that IBs formed by Aβ42 and mutated Htt proteins share a similar pathway for their autophagic clearance.

## Discussion

Autophagy plays a key role in the clearance of misfolded proteins. Misfolded proteins are prone to aggregation, and constant expression of misfolded proteins leads to IB formation. It is well established that aggrephagy selectively clears misfolded protein aggregates [73,74]. Despite our previous studies that implicate autophagy in the clearance of mHtt IBs [34,35], the full scope of IBophagy pathway remains obscure. In this study, by using the protocol that induces autophagic clearance of Htt103QP IBs in budding yeast, we demonstrated that almost all the core autophagy-related genes were essential for this autophagy. We further presented evidence indicating that autophagic clearance of Htt103QP IBs is selective. In addition, we identified several IBophagy SARs, including Atg36, Atg39, and Atg40. Importantly, a similar pathway is required for the autophagic clearance of IBs formed by the Alzheimer’s disease-associated protein Aβ42 in yeast cells. Therefore, our data uncover a new type of autophagy, IBophagy, which is specific for IBs.

Autophagy can be selective or non-selective. Selective autophagy utilises a scaffold protein Atg11 to bridge SAR-associated cargos to the core autophagy machinery for autophagosome formation [20]. We found that Atg11 is required for IBophagy, indicating that IBophagy is selective. Interestingly, deletion of Atg17, the counterpart of Atg11 for non-selective autophagy, resulted in a partial IBophagy defect. Atg17 works in a complex with Atg29 and Atg31 for starvation-induced non-selective autophagy [75], while Atg29 and Atg31 are dispensable for IBophagy. The additional function of both Atg11 and Atg17 in autophagy is the recruitment of Atg9 lipid vesicles to the pre-autophagosomal site for membrane elongation [56]. Therefore, the partial IBophagy defect seen in *atg17Δ* cells could be due to compromised Atg9 vesicle recruitment. In contrast, the Atg29-Atg31 subcomplex inhibits Atg17-dependent Atg9 vesicle recruitment to the autophagosome [56,63,75], which could explain the different requirement of Atg17, Atg29, and Atg31 in IBophagy. Together, these results support the notion that Htt103QP IBophagy is selective.

Because SARs mediate the delivery of specific cargos to the autophagy machinery, we also determined which SARs are required for IBophagy. Previous studies have shown that SAR Cue5/Tollip is implicated in autophagic clearance of misfolded proteins, including mHtt aggregates [26]. Thus, Cue5 is believed to the SAR for aggrephagy. Among the SARs in yeast, Cue5 is the only one that lacks the interaction with Atg11. Consistently, we showed that Atg11, but not Cue5, is required for mHtt IBophagy [20]. Therefore, Cue5 is responsible for aggrephagy that clears misfolded protein aggregates, but not for IBophagy.

From the known yeast SARs, we found Atg36 (pexophagy), Atg39 (nucleophagy/ER-phagy), and Atg40 (ER-phagy) were essential for Htt103QP/Aβ42 IBophagy. Furthermore, Lnp1, an Atg40 cofactor, is required for both ER-phagy and IBophagy. In addition, the Cvt SAR Atg19 is partially required for IBophagy. One thing in common for these SARs is their membrane localisation. Therefore, our speculation is that IBs hijack these membrane-localised SARs for IBophagy. Indeed, cryo-electron tomography has revealed that the surface of mHtt IBs is enriched with ER membrane, endomembrane, and vesicles [16]. We further confirmed that Atg36, Atg39, and Atg40 strongly colocalize and associate with Htt103QP IBs/aggregates. However, it is unclear why mitophagy SAR Atg32 is dispensable for IBophagy, although mitochondria also localise at the mHtt IB periphery [16]. If IBs hijack membrane-localised SARs for IBophagy, it is easy to understand many SARs are required for IBophagy, but more work is needed to clarify why deletion of one of these SARs is sufficient to block IBophagy.

The autophagy pathway is highly conserved from yeast to humans. One open question is whether IBophagy is conserved in human cells. Deletion of core autophagy genes leads to accumulation of polyubiquitinated inclusions/aggregates in mouse neurons [21,22]. Moreover, selective SARs p62, OPTN, and ALFY localise at mHtt aggregates, likely facilitating their autophagic clearance [23-25], but it remains to be determined if these SARs are required for aggrephagy or IBophagy. Our findings suggest that IBophagy requires SARs Atg36, Atg39, and Atg40 in budding yeast. In mammalian cells, NBR1 and P62 act as the SARs for pexophagy [76,77]. For ER-phagy, six receptors, AM134B, RTN3, SEC62, CCPG1, TEX264, and ATL3, have been identified [54,64]. Therefore, further study is needed to analyse if IBophagy occurs in mammalian cells and which SARs play a role in IBophagy.

## Materials and methods

### Strains, plasmids, and growth conditions

Yeast strains used in this study are in W303 background unless otherwise noted. The genotypes of the yeast strains used in this study are listed in Table S1. GFP-tagging of SARs was performed using a PCR-based method [78]. Primers are listed in Table S2. The FLAG- and GFP-tagged Htt103QP fragment with a galactose-inducible promoter (*P_GAL_Flag-Htt103QP-GFP*) was originally from the Lindquist lab [41]. The strain containing GFP-tagged *ATG36* was originally from the Nakatogawa lab [79]. Some strains were constructed utilising the *P_MFA1_-HIS3* selection method. In this method, the *MAT*α parent strain contains a *P_MFA1_-HIS3* reporter, and is crossed with a *MAT*a query strain. This allows for selection of *MAT*a haploid cells after mating and meiosis, as *P_MFA1_-HIS3* is only expressed in *MAT*a cells. The resulting *MAT*a cells are then selected for the desired markers [80,81]. Yeast extract/peptone media supplied with glucose or galactose was used for the growth of yeast strains.

### Detailed protocol for IBophagy induction

For the IBophagy assay, we first grew yeast cells containing *VPH1-mApple pep4∆ P_GAL_-FLAG-Htt103QP-GFP* in YPD (yeast extract-peptone-dextrose) to saturation. Cells were then diluted into galactose media (yeast extract, peptone, galactose) at 1:1000 and incubated for 16 hours at 30°C to induce Htt103QP IB formation. Glucose was then added to 2% to suppress Htt103QP expression, which induces IBophagy. In addition to glucose, we also added 200 mM hydroxyurea to block the cell cycle in S-phase, which eliminates the potential effect of cell division on the percentage of cells with IB or vacuolar GFP. Cells were collected before glucose addition (time 0). After glucose addition, we collected samples every hour for six hours. The collected cells were washed with water, resuspended in 1× PBS, and then immediately subjected to fluorescence imaging.

The YIp351 (pRS304-*P_GAL_α-synuclein-GFP*) plasmid was obtained from the Franco lab. We first linearised the plasmid with MfeI enzyme in the *TRP1* gene, then the plasmid was inserted into the yeast genome after transformation and the subsequent selection on TRP dropout plates. We obtained the Aβ42 plasmid p1771 (pRS416-*P_GAL_Aβ42-GFP*) from the Liebman lab. The *P_GAL_Aβ42-GFP* fragment was subcloned into an integrating plasmid pRS406 with restriction enzymes SacI and KpnI. The resulting plasmid was linearised with XcmI within the *URA3* gene and integrated into the yeast genome by transformation and the subsequent selection on URA dropout plates. The resulting strains were used to examine the autophagy of Aβ42-GFP and α-synuclein-GFP IBs as described for Htt103QP IBophagy.

### Fluorescence imaging and analysis

The analysis of Htt103QP IB formation and autophagic clearance was carried out using a fluorescence microscope (Keyence BZ-X700; Keyence of America, Itasca, IL). Fluorescence signals from the prepared cells were examined under the fluorescence microscope with a 60× objective. Images were taken with appropriate channels for mApple and GFP and z-stacks were taken with a pitch of 0.2 μm. BZ-X700 software was used to create composites by merging the stacked images. Vacuolar GFP intensity was measured in 50 cells per time point using ImageJ. A circle with an area of 4 square pixels was selected inside the vacuole and the average GFP fluorescence intensity inside the area was measured and recorded.

### Western blotting

Protein samples were prepared using an alkaline method and resolved by 10% SDS–PAGE. Anti-FLAG antibody was purchased from Sigma-Aldrich (St. Louis, MO); anti-Myc antibody was from BioLegend (San Diego, CA); anti-Pgk1 antibody was from Invitrogen (Waltham, MA). The horseradish peroxidase–conjugated goat anti-mouse IgG secondary antibody was from Cell Signalling Technology (Danvers, MA).

### Co-immunoprecipitation assay

Cell cultures were grown in raffinose media at 30°C for 16 hours to log phase before treatment with galactose for two hours to induce Htt103QP expression. After being resuspended in RIPA buffer (25 mM Tris, pH 7.5, 10 mM EDTA, 150 mM NaCl, and 0.05% Tween-20) supplied with protease inhibitors, cells were broken with a bead beater. Input sample was collected, and the remaining cell extracts were then incubated with anti-FLAG beads (Sigma-Aldrich; St. Louis, MO) for 2 hours at 4°C. After incubation, the beads were collected by centrifugation and washed three times with RIPA buffer supplied with protease inhibitors. After removal of RIPA buffer, protein loading buffer was added, and the protein samples were boiled twice for 5 min for Western blotting.

### Filter-trap assay

To prepare the samples for the filter-trap assay, we grew yeast cells in galactose media at 30°C for 16 hours to induce Htt103QP IB formation. Cells were collected and washed with wash buffer (10 mM Tris-HCl pH 8.0, 150 mM NaCl, 0.1% SDS) supplied with protease inhibitors. Then, the cells were resuspended in 100 μL of sample buffer (10 mM Tris-HCl pH 8.0, 150 mM NaCl, 2% SDS) supplied with protease inhibitors, and 100 μL of glass beads (0.5 mm) was added. Cells were bead-beaten three times for 20 seconds with one-minute incubations on ice between sessions. Cell debris was cleared by centrifuging at 300 *g* for one minute. Supernatants were collected and 5 μL of 1 M DTT was added. The samples were then boiled for 5 minutes. The filter-trap apparatus (Bio-Dot Apparatus; Bio-Rad, Hercules, CA) was assembled with presoaked filter paper and 0.45 μm nitrocellulose membrane. Wells were washed three times with 100 μL of wash buffer. Samples were then loaded and filtered through the nitrocellulose membrane using a vacuum. Used wells were then washed three times with 100 μL of wash buffer. The membranes were probed with anti-Myc (BioLegend; San Diego, CA) or anti-FLAG (Sigma-Aldrich; St. Louis, MO) antibodies before adding anti-mouse secondary antibody (Cell Signalling Technology; Danvers, MA). In the case of *ATG40-13Myc* and *ATG40-13Myc P_GAL_-FLAG-Htt103QP-GFP* strains, samples were diluted at 1:10 into 2% SDS because of the high protein levels of Atg40 in yeast cells.

### Statistical analysis

Experimental data are expressed as mean ± standard error of the mean (SEM). The fluorescence intensity average at each timepoint was determined by measuring vacuolar GFP intensity using ImageJ in 50 cells for each yeast strain. We then performed statistical analyses using GraphPad software. One-way or two-way ANOVAs were used to determine *p*-values. The exact test is indicated in the figure legends. Statistical significance was determined when *p* < 0.05 (*) and is denoted as such.

## Supplementary Material

Supplemental Material
