# Supplementary material for: Neurodegenerative disease-associated inclusion bodies are cleared by selective autophagy in budding yeast
Source: Autophagy Rep. 2023 Aug 7;2(1):2236407. doi: 10.1080/27694127.2023.2236407 (PMC10482306; doi:10.1080/27694127.2023.2236407)

Figure S1 (Fig. 1)

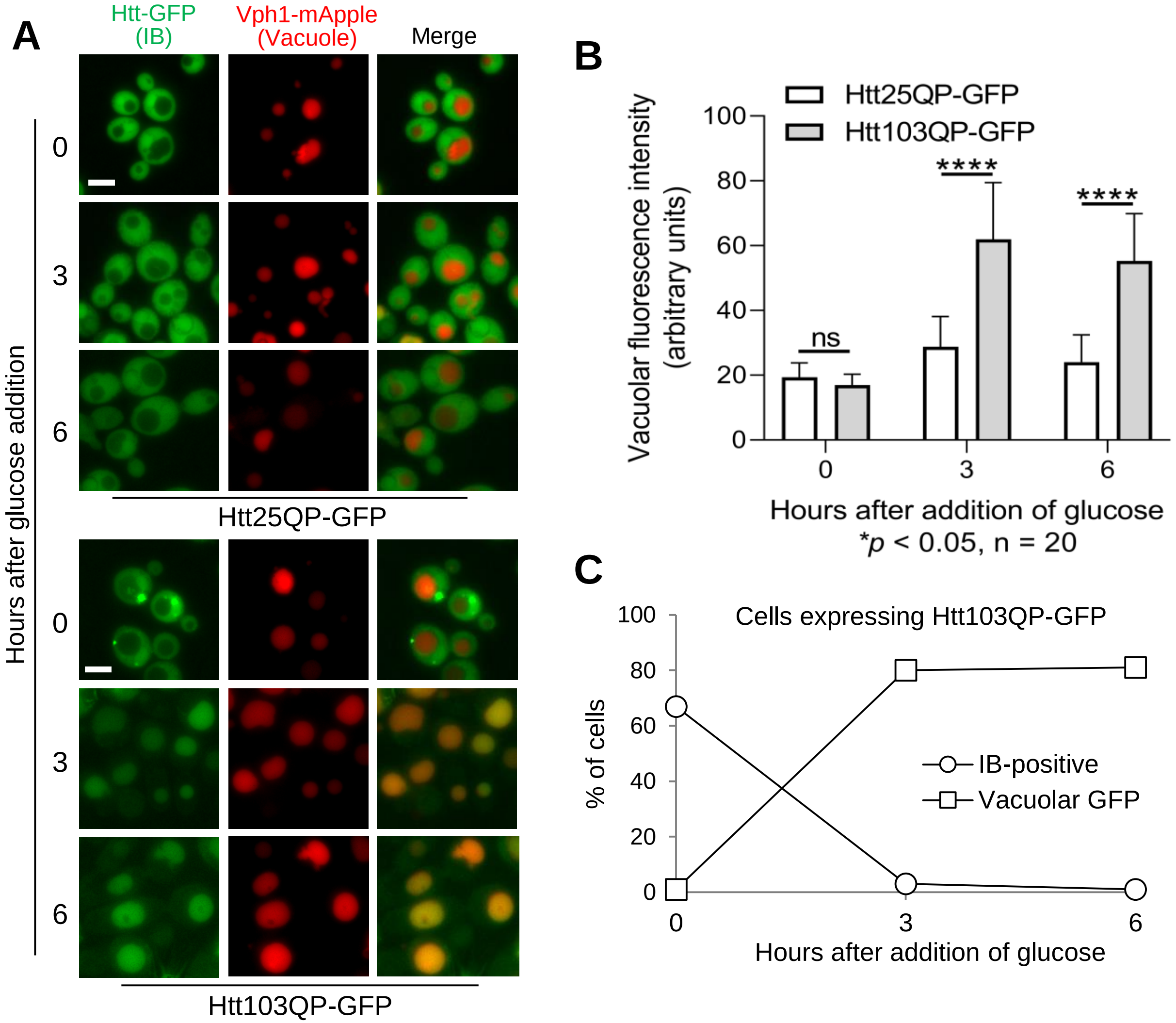

Figure S2 (Fig. 1)

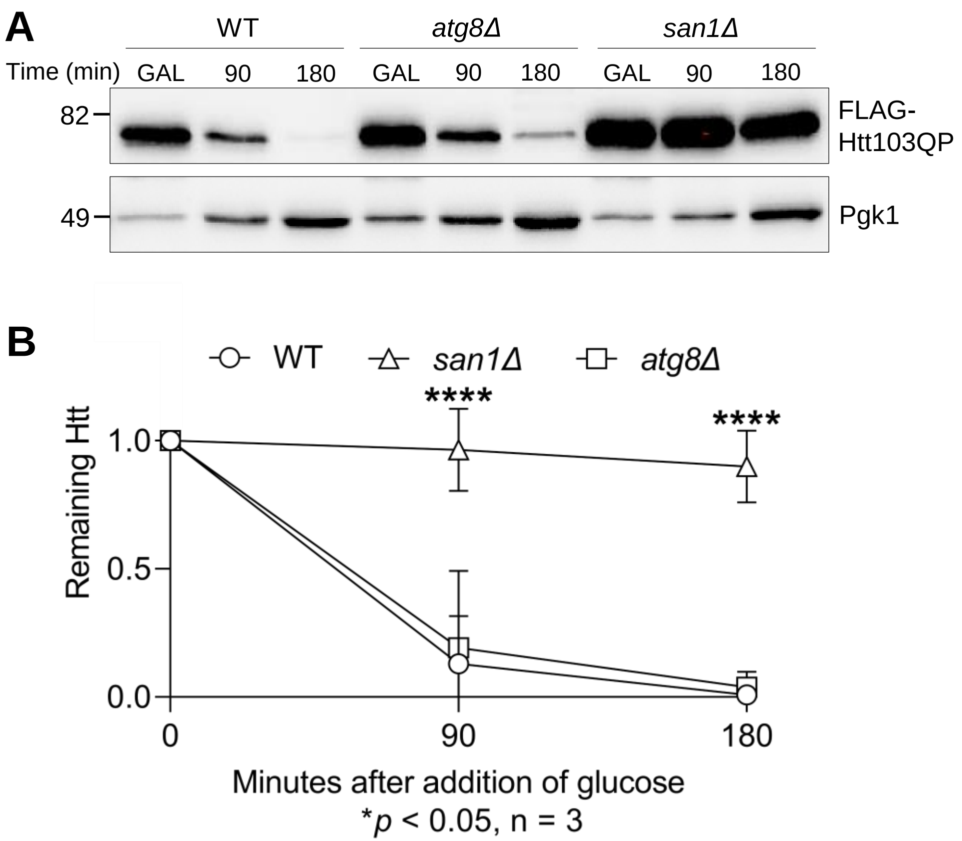

Figure S3 (Fig. 1)

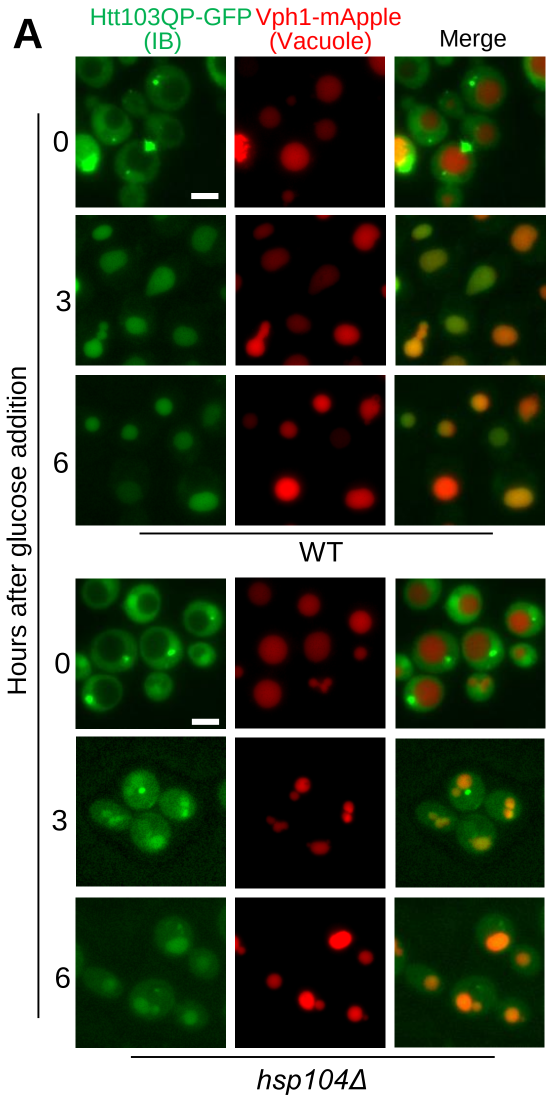

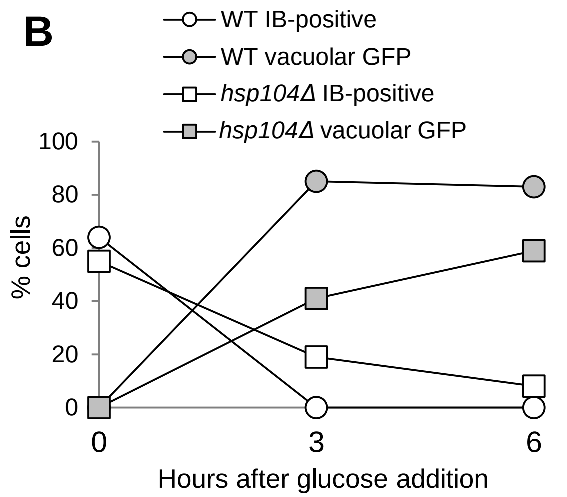

Figure S4 (Fig. 1)

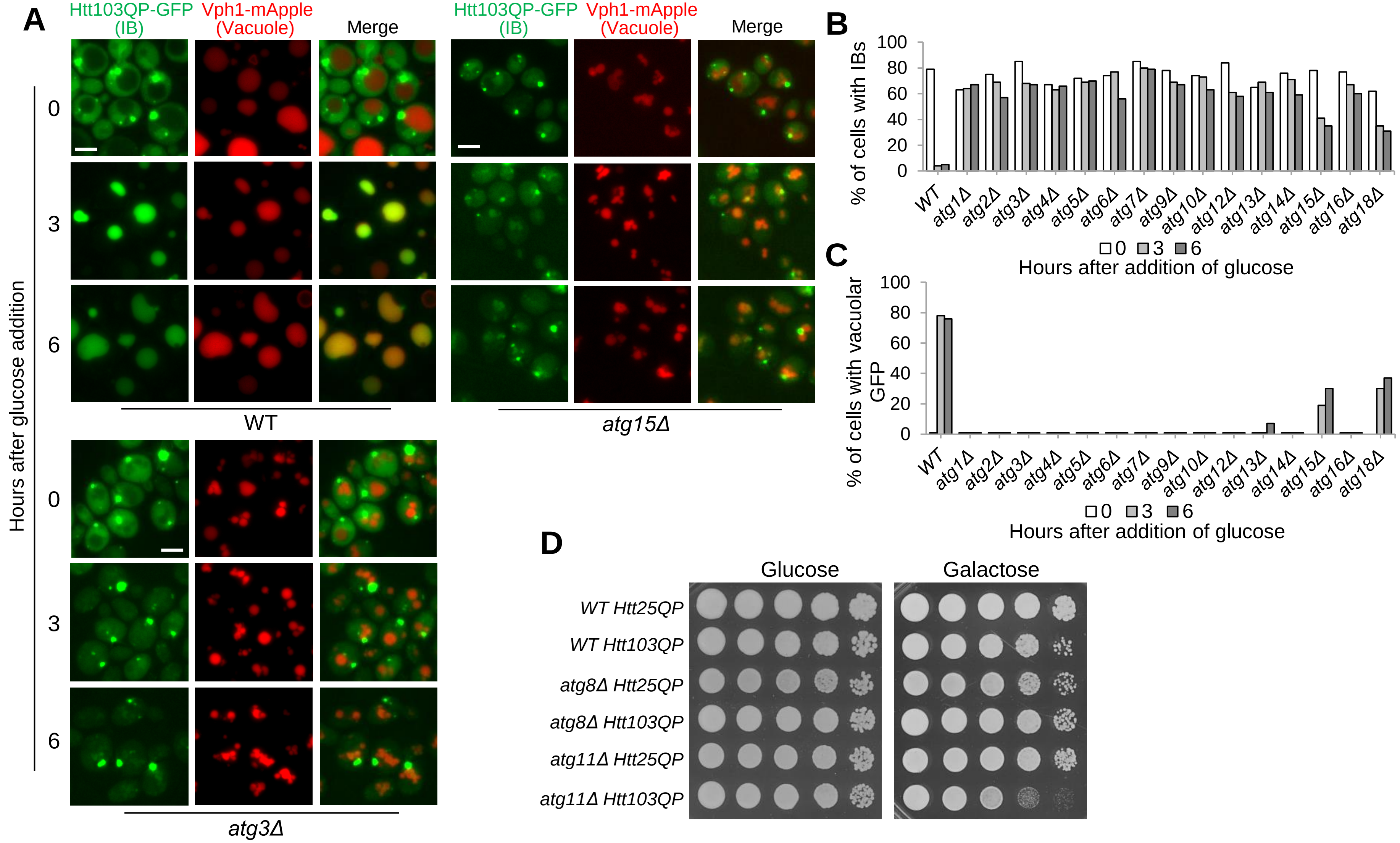

Figure S5 (Fig. 2)

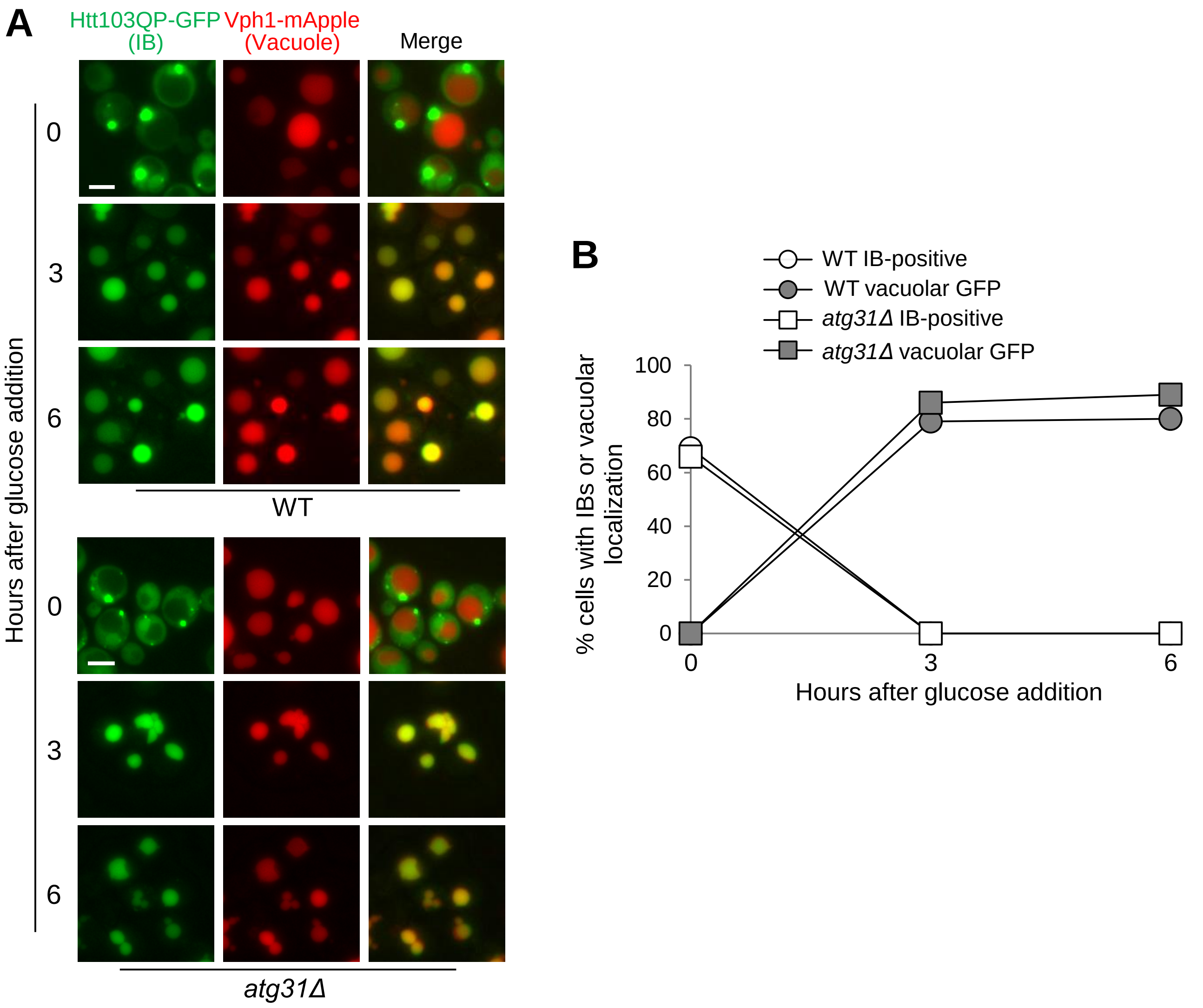

Figure S6 (Fig. 4)

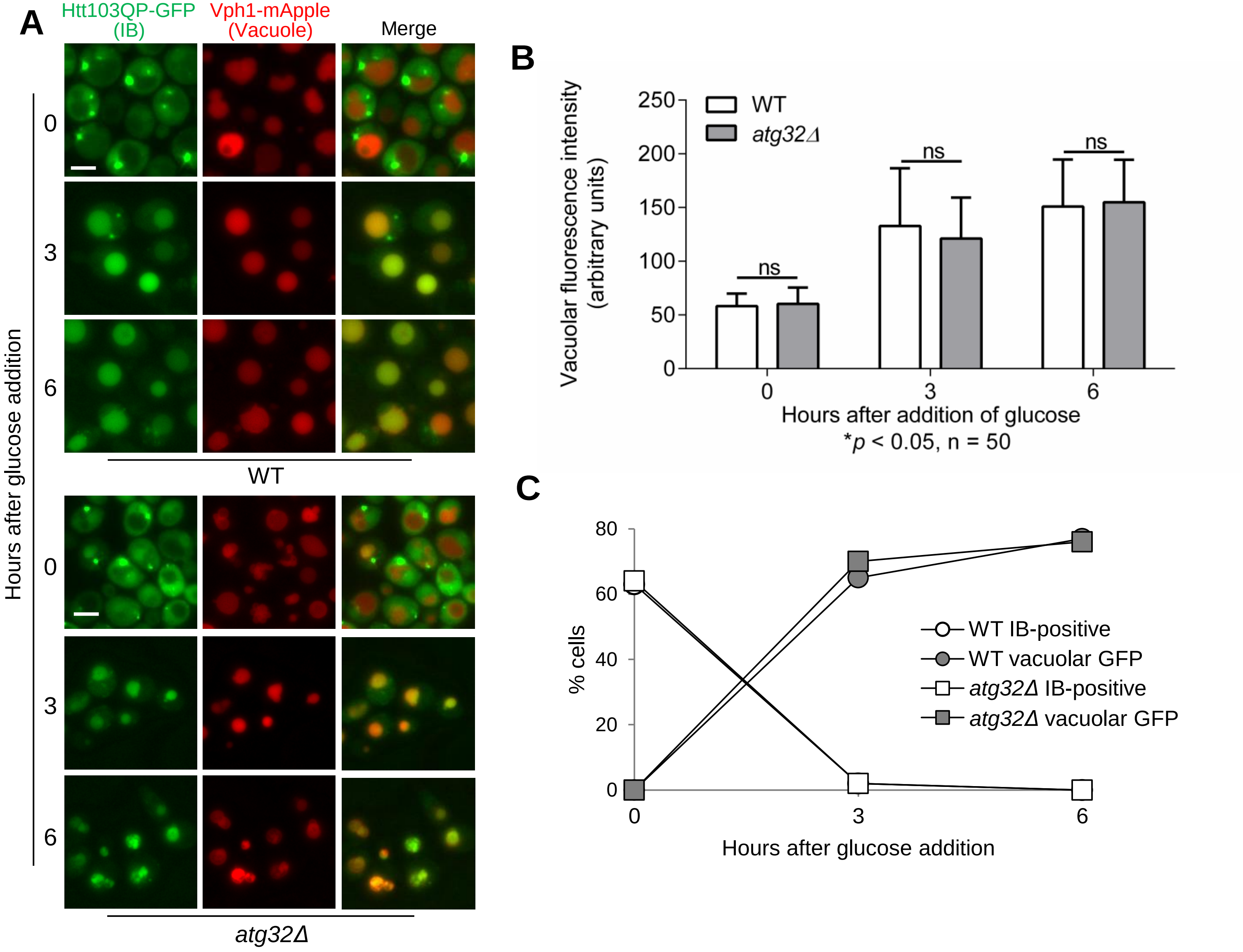

Figure S7 (Fig. 5)

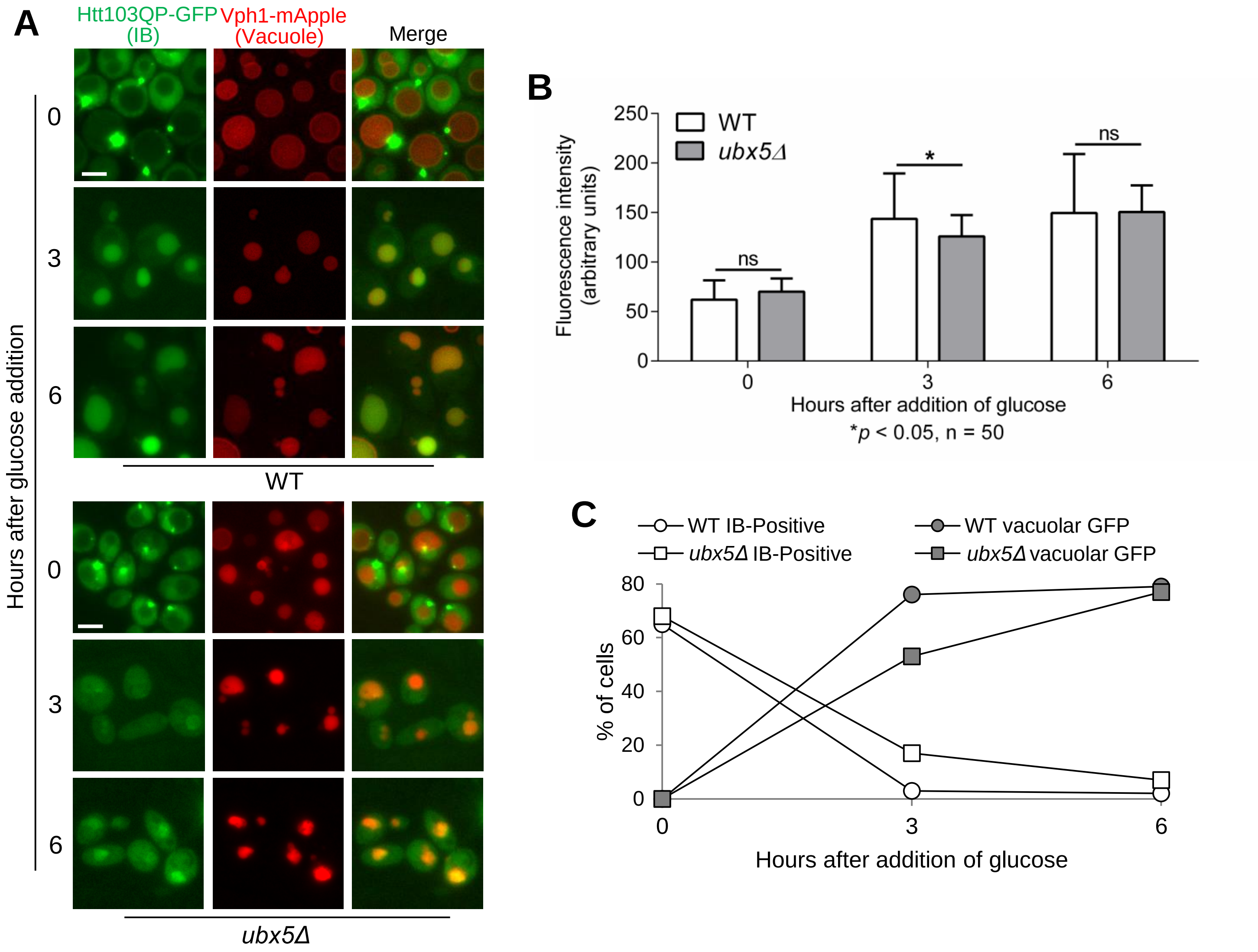

Figure S8 (Fig. 5)

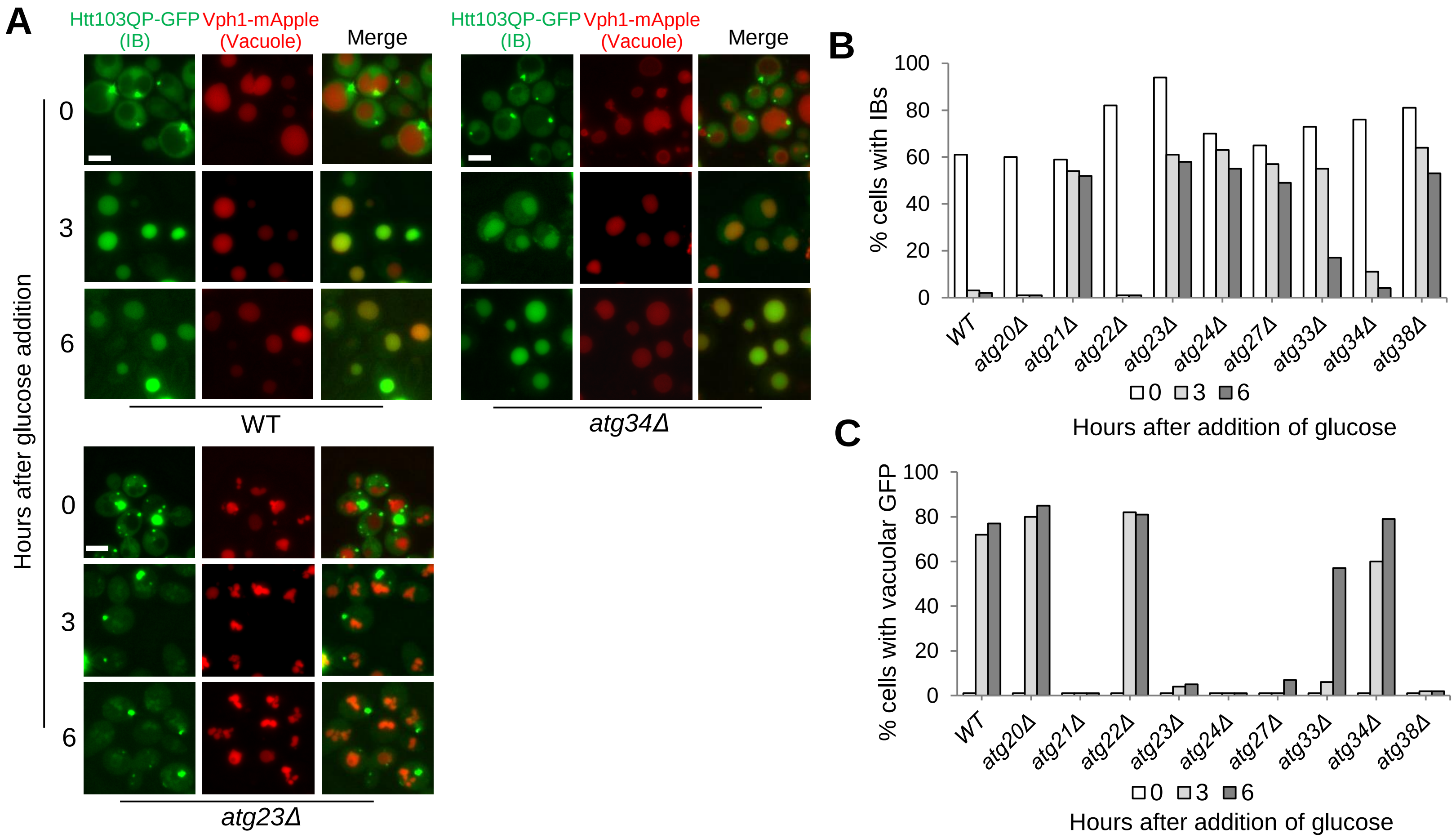

Figure S9 (Fig. 7)

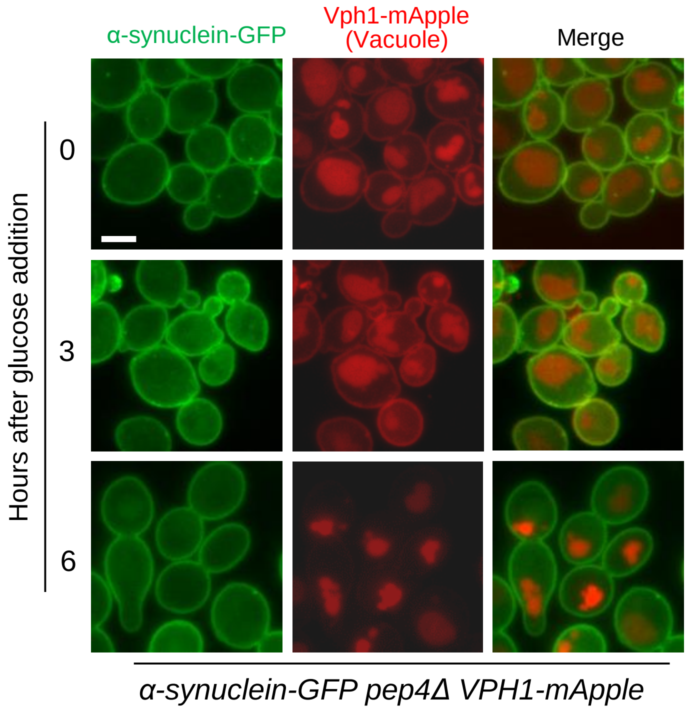

Supplement: Supplemental Material [file KAUO_A_2236407_SM0632.zip › IBophagy Figures resub final sup.pdf]
