# Supplementary material for: Neurodegenerative disease-associated inclusion bodies are cleared by selective autophagy in budding yeast
Source: Autophagy Rep. 2023 Aug 7;2(1):2236407. doi: 10.1080/27694127.2023.2236407 (PMC10482306; doi:10.1080/27694127.2023.2236407)
Supplement: Supplemental Material [file KAUO_A_2236407_SM0632.zip › IBophagy_rev_final_Sup_clean.docx]

**SUPPORTING INFORMATION**

**Figure S1. Trafficking of Htt25QP-GFP and Htt103QP-GFP to the vacuole.** **A)** Vacuole localization of Htt25QP/Htt103QP-GFP after IBophagy induction. Strains containing Htt25QP-GFP and Htt103QP-GFP were grown in galactose media for 16 hours to induce their expression. Glucose was added for IBophagy assay. The localization of Htt25/Htt103QP-GFP and Vph1-mApple (vacuole) before and after IBophagy induction is shown. Scale bar = 5 μm. **B)** Quantification of vacuolar Htt25QP-GFP and Htt103QP-GFP was done by measuring GFP fluorescence intensity inside the vacuole in 50 cells at each time point. Statistical significance was determined by **p* < 0.05, using Sidaks’s two-way ANOVA. **C)** The percentage of cells that contained either an IB or vacuolar GFP signal in the Htt103QP strain over time.

**Figure S2. Proteasomal Htt103QP degradation in an autophagy-deficient mutant.** **A)** Proteasomal degradation of Htt103QP is normal in autophagy defective mutant *atg8Δ*. WT, *atg8Δ*, and *san1Δ* strains were grown to log phase in raffinose media before the addition of galactose to induce Htt103QP expression. After one-hour induction, glucose was added to shut off expression. Samples were collected before (GAL) and after glucose addition (90 and 180 minutes). The protein level of FLAG-Htt103QP-GFP was examined using Western blotting with anti-FLAG antibody. **B)** Blots were quantified by measuring the intensity of protein bands and normalized to the intensity before glucose addition (GAL). Statistical significance was determined by **p* < 0.05, using Tukey’s two-way ANOVA.

**Figure S3. Disaggregase Hsp104 is not required for Htt103QP IBophagy. A)** IBophagy proceeds normally in *hsp104Δ* cells. The IBophagy protocol was the same as described. Here, we show the localization of Htt103QP-GFP and the vacuole (Vph1-mApple) before and after IBophagy induction. Scale bar = 5 μm. **B)** IBophagy was also quantified by counting the percentage of cells that contained either an IB or vacuolar GFP signal (n > 100).

**Figure S4. Htt103QP IBophagy in mutants lacking core autophagy related genes. A)** Autophagy defects were detected in *atg1Δ, atg2Δ, atg4Δ,* *atg5Δ,* *atg6Δ,* *atg7Δ,* *atg9Δ,* *atg10Δ,* *atg12Δ,* *atg13Δ,* *atg14Δ,* *atg15Δ,* *atg16Δ,* and *atg18Δ* mutant cells. The localization of Htt103QP-GFP signal was examined after IBophagy induction. Here, we show the representative images of Htt103QP-GFP and Vph1-mApple (vacuole) before and after IBophagy induction in WT, *atg3Δ* and *atg15Δ* mutants. Scale bar = 5 μm. **B)** IBophagy was quantified by counting the percentage of cells with IBs before (0) and after IBophagy induction (3 and 6 hours) **C)** IBophagy was also quantified by counting the percentage of cells that contained vacuolar GFP signal before and after IBophagy induction (n >100). **D)** The sensitivity of autophagy-deficient mutants to the overexpression of Htt25/Htt103QP-GFP. WT, *atg8Δ,* and *atg11Δ* cells containing *P_GAL_Htt25QP/Htt103QP-GFP* were grown to saturation, 10-fold diluted, and spotted onto glucose (YPD) or galactose (YEPG; yeast extract peptone and galactose) plates. The plates were incubated at 30°C for 2 days before imaging.

**Figure S5. Htt103QP IBophagy in the absence Atg31.** **A)** IBophagy proceeds normally in *atg31Δ* cells. Here, we show the localization of Htt103QP-GFP signal before and after IBophagy induction in WT and *atg31Δ* cells. Vph1-mApple marks the vacuole. Scale bar = 5 μm. **B)** IBophagy was also quantified by counting the percentage of cells that contained either an IB or vacuolar GFP signal (n >100).

**Figure S6. Htt103QP IBophagy in the absence of mitophagy SAR Atg32.** **A)** IBophagy proceeds normally in *atg32Δ* cells. Here, we show the localization of Htt103QP-GFP signal before and after IBophagy induction in WT and *atg32Δ* cells. Vph1-mApple marks the vacuole. Scale bar = 5 μm. **B)** IBophagy was quantified by measuring GFP fluorescence intensity inside the vacuole in 50 cells at each timepoint. Statistical significance was determined by **p* < 0.05, using Sidak’s two-way ANOVA. **C)** IBophagy was also quantified by counting the percentage of cells that contained either an IB or vacuolar GFP signal during IBophagy induction (n >100).

**Figure S7. Htt103QP IBophagy in the absence of Ubx5, the SAR for damaged Cdc48. A)** IBophagy occurs normally in *ubx5Δ* mutant cells. The images were taken to examine the localization of Htt103QP-GFP inside the vacuole (Vph1-mApple) in WT and *ubx5Δ* cells before and after IBophagy induction. Scale bar = 5 μm. **B)** IBophagy was quantified by measuring GFP fluorescence intensity inside the vacuole in 50 cells at each timepoint. Statistical significance was determined by **p* < 0.05, using Sidak’s two-way ANOVA. **C)** IBophagy was also quantified by counting the percentage of cells that contained either an IB or vacuolar GFP signal.

**Figure S8. The requirement of components in the Cvt pathway for Htt103QP IBophagy. A)** Htt103QP IBophagy in mutants lacking Cvt components. Images were taken before and after IBophagy induction to examine vacuole localization of Htt103QP-GFP in WT, *atg23Δ,* and *atg34Δ* cells. The vacuole is marked with Vph1-mApple. Scale bar = 5 μm. **B)** IBophagy was quantified by counting the percentage of cells with IBs before (0) and after IBophagy induction (3 and 6 hours). **C)** IBophagy was also quantified by counting the percentage of cells that contained vacuolar GFP signal before and after IBophagy induction (n >100).

**Figure S9. IBophagy in yeast cells expressing α-synuclein.** Strains containing *P_GAL_α-synuclein-GFP* were grown in galactose media for 16 hours before being subjected IBophagy assay. The images were taken to examine the localization of α-synuclein-GFP and the vacuole (Vph1-mApple) before and after IBophagy induction. Scale bar = 5 μm.

**Table S1. Yeast strains used in this study**

| **Strains** | **Genotype** | **Reference** |
| --- | --- | --- |
| Y300 | Mat**a** *ura3-1, his3-11,15 leu2-3,112 trp1-1, ade2-1, can1-100* | Lab Stock |
| 3216-1-1 | Mat**a** *pep4Δ::KanMX VPH1-mApple-Sphis5^+^ P_GAL_FLAG-Htt103QP-GFP-URA3* | Lab stock |
| 4102-3-2 | Mat**α** *atg8Δ::KanMX pep4Δ::NAT VPH1-mApple-Sphis5^+^ P_GAL_FLAG-103QP-GFP-URA3* | Lab stock |
| 4358-2-1 | Mat**a** *atg11Δ::KanMX pep4Δ::NAT VPH1-mApple-Sphis5^+^ P_GAL_FLAG-103QP-GFP-URA3* | This study |
| 4357-3-1 | Mat**a** *atg17Δ::KanMX pep4Δ::NAT VPH1-mApple-Sphis5^+^ P_GAL_FLAG-103QP-GFP-URA3* | This study |
| 3833-3-1 | Mat**a** *atg29Δ::KanMX pep4Δ::NAT VPH1-mApple-Sphis5^+^ P_GAL_FLAG-103QP-GFP-URA3* | Lab stock |
| 3950-10-1 | Mat**α** *cue5Δ::KanMX pep4Δ::NAT VPH1-mApple-Sphis5^+^ P_GAL_FLAG-103QP-GFP-URA3* | Lab stock |
| 4093-7-1 | Mat**a** *atg36Δ::KanMX pep4Δ::NAT VPH1-mApple-Sphis5^+^ P_GAL_FLAG-103QP-GFP-URA3* | This study |
| 4088-3-1 | Mat**a** *atg39Δ::KanMX pep4Δ::NAT VPH1-mApple-Sphis5^+^ P_GAL_FLAG-103QP-GFP-URA3* | This study |
| 4089-1-1 | Mat**α** *atg40Δ::KanMX pep4Δ::NAT VPH1-mApple-Sphis5^+^ P_GAL_FLAG-103QP-GFP-URA3* | This study |
| 4099-7-1 | Mat**a** *lnp1Δ::KanMX pep4Δ::NAT VPH1-mApple-Sphis5^+^ P_GAL_FLAG-103QP-GFP-URA3* | This study |
| 4096-1-4 | Mat**α** *pex3Δ::KanMX pep4Δ::NAT VPH1-mApple-Sphis5^+^ P_GAL_-FLAG-103QP-GFP-URA3* | This study |
| 4464-3-1 | Mat**a** *ATG36-GFP-KanMX P_GAL_FLAG-Htt103QP-mApple-URA3 snf7::KanMX* | This study |
| 4431-3-1 | Mat**a** *ATG39-GFP- Sphis5^+^ P_GA_LFLAG-Htt103QP-mApple-URA3 snf7::KanMX* | This study |
| 4432-4-2 | Mat**α** *ATG40-GFP- Sphis5^+^ P_GAL_FLAG-Htt103QP-mApple-URA3 snf7::KanMX* | This study |
| 4107-1-4 | Mat**α** *atg19Δ::KanMX pep4Δ::NAT VPH1-mApple-Sphis5^+^ P_GAL_FLAG-103QP-GFP-URA3* | This study |
| 3829-1-4 | Mat**a** *atg32Δ::KanMX pep4Δ::NAT VPH1-mApple-Sphis5^+^ P_GAL_FLAG-103QP-GFP-URA3* | This study |
| 4460-1-3 | Mat**a** *atg15Δ::KanMX pep4Δ::NAT VPH1-mApple-Sphis5^+^ P_GAL_FLAG-103QP-GFP-URA3* | This study |
| 4074-13-1 | Mat**a** *ubx5Δ::KanMX pep4Δ::NAT VPH1-mApple-Sphis5^+^ P_GAL_FLAG-103QP-GFP-URA3* | This study |
| 4508-5-1 | Mat**α** *ATG40-GFP-HIS3 P_GAL_FLAG-Htt103QP-mApple-URA3 snf7::KanMX lnp1Δ::KanMX* | This study |
| 4151-5-3 | Mat**a** *atg31Δ::KanMX pep4Δ::NAT VPH1-mApple-Sphis5^+^ P_GAL_FLAG-103QP-GFP-URA3* | This study |
| 3264-1-2 | Mat**α** *atg1Δ::KanMX pep4Δ::NAT VPH1-mApple-Sphis5^+^ P_GAL_FLAG-103QP-GFP-URA3* | Lab stock |
| *atg2Δ* | Mat**a** *atg2Δ::KanMX pep4Δ::NAT mfa1:P_MFA1_Sphis5^+^ P_GAL_FLAG-103QP-GFP-URA3* | This study |
| *atg3Δ* | Mat**a** *atg3Δ::KanMX pep4Δ::NAT mfa1:P_MFA1_Sphis5^+^ P_GAL_FLAG-103QP-GFP-URA3* | This study |
| 4565-1-4 | Mat**a** *atg4Δ::KanMX pep4Δ::KanMX VPH1-mApple-Sphis5^+^ P_GAL_FLAG-103QP-GFP-URA3* | This study |
| *atg5Δ* | Mat**a** *atg5Δ::KanMX pep4Δ::NAT mfa1:P_MFA1_Sphis5^+^ P_GAL_FLAG-103QP-GFP-URA3* | This study |
| *atg6Δ* | Mat**a** *atg6Δ::KanMX pep4Δ::NAT mfa1:P_MFA1_Sphis5^+^ P_GAL_FLAG-103QP-GFP-URA3* | This study |
| *atg7Δ* | Mat**a** *atg7Δ::KanMX pep4Δ::NAT mfa1:P_MFA1_Sphis5^+^ P_GAL_FLAG-103QP-GFP-URA3* | This study |
| *atg9Δ* | Mat**a** *atg9Δ::KanMX pep4Δ::NAT mfa1:P_MFA1_Sphis5^+^ P_GAL_FLAG-103QP-GFP-URA3* | This study |
| *atg10Δ* | Mat**a** *atg10Δ::KanMX pep4Δ::NAT mfa1:P_MFA1_Sphis5^+^ P_GAL_FLAG-103QP-GFP-URA3* | This study |
| *atg12Δ* | Mat**a** *atg12Δ::KanMX pep4Δ::NAT mfa1:P_MFA1_Sphis5^+^ P_GAL_FLAG-103QP-GFP-URA3* | This study |
| *atg13Δ* | Mat**a** *atg13Δ::KanMX pep4Δ::NAT mfa1:P_MFA1_Sphis5^+^ P_GAL_FLAG-103QP-GFP-URA3* | This study |
| *atg14Δ* | Mat**a** *atg14Δ::KanMX pep4Δ::NAT mfa1:P_MFA1_Sphis5^+^ P_GAL_FLAG-103QP-GFP-URA3* | This study |
| *atg16Δ* | Mat**a** *atg16Δ::KanMX pep4Δ::NAT mfa1:P_MFA1_Sphis5^+^ P_GAL_FLAG-103QP-GFP-URA3* | This study |
| 4578-4-4 | Mat**a** *atg18Δ::KanMX pep4Δ::NAT VPH1-mApple-Sphis5^+^ P_GAL_FLAG-103QP-GFP-URA3* | This study |
| 3838-11-2 | Mat**a** *atg20Δ::KanMX pep4Δ::NAT VPH1-mApple-Sphis5^+^ P_GAL_FLAG-103QP-GFP-URA3* | This study |
| 3825-1-4 | Mat**a** *atg21Δ::KanMX pep4Δ::NAT VPH1-mApple-Sphis5^+^ P_GAL_FLAG-103QP-GFP-URA3* | This study |
| 3845-2-2 | Mat**α** *atg22Δ::KanMX pep4Δ::NAT VPH1-mApple-Sphis5^+^ P_GAL_FLAG-103QP-GFP-URA3* | This study |
| *atg23Δ* | Mat**a** *atg23Δ::KanMX pep4Δ::NAT mfa1:P_MFA1_Sphis5^+^ P_GAL_FLAG-103QP-GFP-URA3* | This study |
| *atg24Δ* | Mat**a** *atg24Δ::KanMX pep4Δ::NAT mfa1:P_MFA1_Sphis5^+^ P_GAL_FLAG-103QP-GFP-URA3* | This study |
| 3832-1-4 | Mat**α** *atg27Δ::KanMX pep4Δ::NAT VPH1-mApple-Sphis5^+^ P_GAL_FLAG-103QP-GFP-URA3* | This study |
| *atg33Δ* | Mat**a** *atg33Δ::KanMX pep4Δ::NAT mfa1:P_MFA1_Sphis5^+^ P_GAL_FLAG-103QP-GFP-URA3* | This study |
| 4108-3-1 | Mat**α** *atg34Δ::KanMX pep4Δ::NAT VPH1-mApple-Sphis5^+^ P_GAL_FLAG-103QP-GFP-URA3* | This study |
| 3837-1-4 | Mat**a** *atg38Δ::KanMX pep4Δ::NAT VPH1-mApple-Sphis5^+^ P_GAL_FLAG-103QP-GFP-URA3* | This study |
| YAF38 | Mat**a** *ATG36-13myc-Sphis5^+^* | This study |
| 4596-3-1 | Mat**a** *ATG36-13myc-Sphis5^+^P_GAL_FLAG-Htt103QP-GFP-URA3* | This study |
| YAF16 | Mat**a** *ATG39-13myc-Sphis5^+^* | This study |
| 4592-4-2 | Mat**a** *ATG39-13myc-Sphis5^+^P_GAL_FLAG-Htt103QP-GFP-URA3* | This study |
| YAF47 | Mat**a** *ATG40-13myc-Sphis5^+^* | This study |
| YAF48 | Mat**a** *ATG40-13myc-Sphis5^+^P_GAL_FLAG-Htt103QP-GFP-URA3* | This study |
| 4601-2-4 | Mat**a** *P_GAL_-Aβ42-eGFP-URA3 pep4Δ::NAT VPH1-mApple-Sphis5^+^* | This study |
| 4602-1-1 | Mat**a** *P_GAL_-α-synuclein-GFP-TRP1 pep4Δ::NAT VPH1-mApple-Sphis5^+^* | This study |
| YAF49 | Mat**α** *atg8Δ::KanMX P_GAL_Aβ42-eGFP-URA3 pep4Δ::NAT VPH1-mApple-Sphis5^+^* | This study |
| 4612-4-1 | Mat**a** *atg11Δ::KanMX P_GAL_Aβ42-eGFP-URA3 pep4Δ::NAT VPH1-mApple-Sphis5^+^* | This study |
| 4626-1-1 | Mat**a** *atg29Δ::KanMX P_GAL_Aβ42-eGFP-URA3 pep4Δ::NAT VPH1-mApple-Sphis5^+^* | This study |
| 4624-1-4 | Mat**α** *cue5Δ::Sphis5^+^ P_GAL_Aβ42-eGFP-URA3 pep4Δ::NAT VPH1-mApple-Sphis5^+^* | This study |
| 4616-2-3 | Mat**α** *atg36Δ::KanMX P_GAL_Aβ42-eGFP-URA3 pep4Δ::NAT VPH1-mApple-Sphis5^+^* | This study |
| 4617-4-1 | Mat**α** *atg39Δ::KanMX P_GAL_Aβ42-eGFP-URA3 pep4Δ::NAT VPH1-mApple-Sphis5^+^* | This study |
| 4618-1-2 | Mat**a** *atg40Δ::KanMX P_GAL_Aβ42-eGFP-URA3 pep4Δ::NAT VPH1-mApple-Sphis5^+^* | This study |
| 4082-1-2 | Mat**a** *san1Δ::KanMX P_GAL_Htt103QP-GFP-URA3 pep4Δ::NAT VPH1-mApple-Sphis5^+^* | lab stock |
| 4575-2-1 | Mat**α** *pep4Δ::Sphis5^+^ VPH1-mApple-Sphis5^+^ P_GAL_FLAG-Htt25QP-GFP-URA3* | This study |
| 4610-1-2 | Mat**α** *atg8Δ::KanMX pep4Δ::Sphis5^+^ VPH1-mApple-Sphis5^+^ P_GAL_FLAG-Htt25QP-GFP-URA3* | This study |
| 4582-2-2 | Mat**a** *atg11Δ::KanMX pep4Δ::Sphis5^+^ VPH1-mApple-Sphis5^+^ P_GAL_FLAG-Htt25QP-GFP-URA3* | This study |
| 4358-6-3 | Mat**a** *atg11Δ::KanMX pep4Δ::NAT VPH1-mApple-Sphis5^+^ P_GAL_FLAG-Htt103QP-GFP-URA3* | This study |
| 4563-2-2 | Mat**a** *hsp104Δ::KanMX pep4Δ::NAT VPH1-mApple-Sphis5^+^ P_GAL_FLAG-Htt103QP-GFP-URA3* | This study |

| **Name** | **Sequence** | **Reference** |
| --- | --- | --- |
| Atg36-Myc13 tag forward primer | TGGCTGGACTTTACTTTCTAGGTTTTTGGACA  GAGAATGGCGGATCCCCGGGTTAATTAA | This study |
| Atg36-Myc13 tag forward primer | GTTCGGACAACGTTTTAGAATGAGGGTATC  TAACTTTCTTGAATTCGAGCTCGTTTAAAC | This study |
| Atg39-GFP/Myc13 tag forward primer | GTCAGAATGCAGGAAAAAAAATTATTTTAAGTG  CAAACACCGGATCCCCGGGTTAATTAA | This study |
| Atg39-GFP/Myc13 tag reverse primer | CTTTTGTTAATTTCATTCTTCATGCTGGGTTTT GGATGATGAATTCGAGCTCGTTTAAAC | This study |
| Atg40-GFP/Myc13 tag forward primer | TTTTATGGAGGATATTCTAGATGAGACAACTGA ATTGGATCGGATCCCCGGGTTAATTAA | This study |
| Atg40-GFP/Myc13 tag reverse primer | CCTTCATAGACTACCATTATGGTAAAATGGAAA AACTATTGAATTCGAGCTCGTTTAAAC | This study |

**Table S2. Oligonucleotides used in this study**
